# Downregulation of miR-760 Causes Human Intervertebral Disc Degeneration by Targeting the MyD88/Nuclear Factor-Kappa B Signaling Pathway

**DOI:** 10.3389/fbioe.2022.813070

**Published:** 2022-04-11

**Authors:** Xueliang Cui, Yanan Li, Junping Bao, Kun Wang, Xiaotao Wu

**Affiliations:** ^1^ Medical School of Southeast University, Nanjing, China; ^2^ Department of Orthopaedics, Zhongda Hospital, Southeast University, Nanjing, China; ^3^ Department of Orthopaedics, Qingdao Women and Children’s Hospital, Qingdao, China

**Keywords:** miR-760, intervertebral disc degeneration, MyD88, nucleus pulposus cells, NF-κB signaling pathway

## Abstract

Dysregulation of microRNAs (miRNAs) plays a critical role in the development of intervertebral disc degeneration (IDD). In this study, we present evidence from *in vitro* and *in vivo* research to elucidate the mechanism underlying the role of miR-760 in IDD. miRNA microarray and quantitative reverse transcription-polymerase chain reaction were used to determine the miRNA profiles in patients with IDD. Functional analysis was performed to evaluate the role of miR-760 in the pathogenesis of IDD. Luciferase reporter and western blotting assays were used to confirm the miRNA targets. The expression of miR-760 was significantly decreased in degenerative nucleus pulposus (NP) cells and negatively correlated with disc degeneration grade. Functional assays demonstrated that miR-760 delivery significantly increased NP cell proliferation and promoted the expression of collagen II and aggrecan. Moreover, *MyD88* was identified as a target gene of miR-760. miR-760 effectively suppressed MyD88 expression by interacting with the 3′-untranslated region, which was abolished by miR-760 binding site mutations. An *in vivo* experiment using an IDD mouse model showed that the upregulation of miR-760 could effectively suspend IDD. Therefore, miR-760 was found to play an important role in IDD and can be used as a promising therapeutic target for the treatment of patients with IDD.

## Introduction

Low back pain (LBP), the leading cause of disability, affects approximately 80% of the global population in their life span (Chenot et al., 2017). The annual economic cost related to LBP is as high as $90 billion in the United States alone (Dieleman et al., 2016). LBP can be caused by a variety of factors, of which intervertebral disc (IVD) degeneration (IDD) accounts for up to 40% of cases (Global Burden of Disease Study 2013 Collaborators, 2015). IVD is a flexible joint located between neighboring vertebral bodies. The main components of IVD are the outer endplates, inner annulus fibrosus (AF), and central nucleus pulposus (NP) (Roberts et al., 2006). NP cells play a crucial role in maintaining the integrity and viscoelastic properties of IVD by producing a complex extracellular matrix (ECM), which is mainly composed of type II collagen and aggrecan (Wang et al., 2013). Degeneration usually starts with NP cells that are replaced by smaller fibrochondrocyte-like cells, resulting in structural collapse and major changes in disc function (Lv et al., 2014). Although multiple factors, including aging, genetics, autoimmunity, lifestyle, and a predisposition to injury have been demonstrated to induce microenvironmental changes in IVD, the precise pathogenesis of IDD remains elusive (Nakamichi et al., 2016). Thus, it is necessary to enhance the understanding of the molecular mechanisms of IDD in order to identify new approaches for targeted therapy.

Recent studies have revealed that microRNAs (miRNAs) play important roles in IDD (Cazzanelli and Wuertz-Kozak, 2020). miRNAs are a class of small and non-coding RNA molecules that mediate their biological functions by binding to the 3′-untranslated region (UTR) of target mRNAs, resulting in either mRNA degradation or translation inhibition (Chen et al., 2014; Papaioannou et al., 2015). A growing body of evidence indicates that aberrant miRNA expression is tightly associated with various aspects of IDD, such as NP cell proliferation (Liu et al., 2014), apoptosis (Wang et al., 2015), inflammatory response (Gu et al., 2015) and the extracellular matrix regeneration (Jing and Jiang, 2015). Some dysregulated miRNAs, such as miR-141, miR-640 and miR-155, have been detected in IDD patients and identified as targets of IDD-associated gene expression ranging from matrix-degrading enzymes to proinflammatory cytokines (Wang et al., 2011; Ji et al., 2018; Dong et al., 2019). In addition, the expression of miRNAs is tissue-specific, suggesting that miRNAs may contribute to the specification and maintenance of tissue identity. Thus, it is necessary to establish a miRNA expression profile to investigate their expression patterns and underlying functional mechanisms that may aid in identifying novel therapeutic targets for IDD.

In this study, we compared the miRNA profiles between patients with IDD and healthy controls using a microarray. Several IDD-specific miRNAs were identified, and miR-760 expression was dramatically downregulated in degenerative NP cells. The downregulation of miR-760 was negatively correlated with the degree of IDD. Furthermore, *in vitro* experiments indicated that miR-760 inhibited IDD by targeting the MyD88/nuclear factor-kappa B (NF-κB) signaling pathway. These data suggest that miR-760 may be a potential target for therapeutic intervention in IDD.

## Methods

### Ethics Statement

The study protocol was approved by the Ethics Committee of the Affiliated Zhongda Hospital of Southeast University (registered number: 2017ZDKYSB095), and written informed consent was obtained from all participants. Human NP samples were obtained from patients undergoing discectomy following the ethical guidelines of the Ethics Committee of the Affiliated Zhongda Hospital of Southeast University.

### Patient Samples

Human NP samples were obtained from 50 patients (mean age 58.1 years; 22 males and 28 females) with degenerative disc disease who underwent discectomy. Indications for discectomy included failure in conservative treatment and progressive neurologic deficits, such as progressive motor weakness or the cauda equina syndrome. Fifteen of the 50 samples were obtained from the level of L4/L5, 35 from L5/S1.

Control samples were collected from 43 patients (mean age 55.6 years; 21 males and 22 females) with fresh traumatic lumbar fractures who underwent anterior decompressive surgery due to neurological deficits. Human NP samples were classified as degenerative disc disease based on routine magnetic resonance imaging (MRI) scans of the lumbar spine. The degree of disc degeneration was graded from T2-weighted images using the Pfirrmann classification (Pfirrmann et al., 2001).

### Mice Model

Wild-type (WT) C57BL/6 mice were purchased from the Animal Center of the Jackson Laboratory (Bar Harbor, ME, United States). WT C57BL/6 (12-week-old) male mice were administered ketamine (100 mg/kg) for general anesthesia. We performed a sagittal skin incision from the sixth coccygeal vertebra (Co6) to the eighth coccygeal vertebra (Co8) to locate the position for needle insertion. Subsequently, in the areas between the sixth and seventh coccygeal vertebrae (Co6–Co7), coccygeal discs were punctured using a syringe needle. To ensure degeneration, we punctured AF along the vertical direction and then rotated the needle in the axial direction by 180° and held it for 10 s. The puncture was made using a 31-G needle, which was inserted 1.5 mm into the disc, to depressurize the nucleus.

For miR-760 treatment of experimental IDD, twelve mice were randomly divided into four groups with different treatments. AgomiR NC, agomiR-760, antagomiR NC, and antagomiR-760 were acquired from IBSBio (Shanghai, China). Ten microlitres of agomiR-760, antagomiR-760, and corresponding negative controls were injected into Co6–Co7 disc of the mice disc using a micro-syringe (Hamilton). All mice were housed under standard diurnal light/dark conditions, fed a standard commercial diet and allowed free access to water. All efforts were made to minimize suffering. The mice received the injection at 3 days, 1, 2, and 3 weeks after IDD surgery. Mice were sacrificed 12 weeks after treatment and subjected to further histopathological and radiographic analyses.

AgomiR-760, antagomiR-760, and corresponding negative controls labeled with Cy3 using the Silencer^®^ siRNA Labeling Kit (#AM1636) were packed in a NP carrier system (MaxSuppressor *In Vivo* RNALANCEr II Kit and Lipid Extruder, BIOO Scientific) according to the manufacturer’s instructions. Based on this, Cy3-agomiR-760, Cy3-antagomiR-760, Cy3-agomiR NC, Cy3-antagomiR NC was obtained. To determine the Transfection efficiency of agomiR-760, antagomiR-760, and corresponding negative controls labeled with Cy3, *in vivo* fluorescence imaging using an IVIS 200 Imaging system (Xenogen, Caliper Life Science, MA, United States) were performed at different time points post-injection (24 and 72 h) in each group. All animal procedures were approved by the Ethics Committee of the Southeast University.

### Histological and Radiographic Evaluation

The discs from the mice were fixed in formalin and embedded in paraffin. The discs were sectioned into pieces with 5 μm thickness and then stained with hematoxylin, eosin, and Safranin O-fast green. Images were taken using an Olympus BX51 microscope (Olympus Center Valley, PA, United States). Radiological evaluation was performed using the disc height index (DHI) (Han et al., 2008) by X-ray examination 12 weeks post-surgery. Measurements of the punctured discs and their corresponding internal control discs were carried out together. IVD height and the adjacent vertebrae heights were measured on the midline and 25% of the disc’s width form the midline on either side. The DHI was defined as the average of the three measurements from midline to the boundary of the central 50% of the disc width divided by the average of the two adjacent vertebrae heights. The DHI changes of punctured discs were expressed as a percentage (%DHI = post-punctured DHI/pre-punctured DHI × 100). Histological scores were obtained using the method described by Masuda et al. (Masuda et al., 2005). Two independent reviewers performed the evaluation, and we took the average of the two as the final scores. The degree of disc degeneration was negatively correlated with DHI%, but positively correlated with histological score.

### Extraction and Primary Culture of Human NP Cells

Human NP tissues obtained from one donor in the control group were first washed with phosphate-buffered saline (PBS) until they were clean, separated from the AF using a stereotaxic microscope, and cut into pieces (2–3 mm^3^). NP cells were released from NP tissues by incubation with 0.25 mg/ml type II collagenase (Invitrogen, Carlsbad, CA, United States) for 12 h at 37°C in Dulbecco’s minimal essential medium/Ham’s F12 (DMEM-F12) medium (Gibco, Grand Island, NY, United States). Mononuclear cells were isolated by passing the tissue through a 200-mesh cell strainer, followed by 80%/40% Percoll gradient centrifugation. After isolation, NP cells were resuspended in DMEM containing 10% fetal bovine serum (FBS) (Gibco), 100 μg/ml streptomycin (Gibco), 100 U/ml penicillin, and 1% glutamine at 37°C incubator with 5% CO_2_. The culture medium was changed every 72 h. When the cell density reached 80%, the cells were detached with 0.25% of trypsin and passaged at a ratio of 1:2 (Ji et al., 2018). No difference in morphology was found between the two generations of cells. Then, the cells (passage two) were used for subsequent experiments. Cells were incubated at a density of 2 × 10^5^ cells per well in a 24-well plate and cultured in DMEM/F12-based culture medium containing 10% FBS and 100 μg/ml streptomycin, 100U/mL penicillin at 37°C in a 5% CO_2_ (v/v) incubator.

### RNA Isolation, cDNA Synthesis, and Quantitative Reverse Transcription-Polymerase Chain Reaction

In *ex vivo*, the human AF and NP tissues were carefully separated and then cut into small pieces (1–3 mm^3^) with a scalpel under the microscope. Then, samples were snap frozen in liquid nitrogen within 40 min of removal from the patient and stored at −80°C until RNA extraction. The extraction of total RNA was achieved with TRIzol (Ambion, Life Technologies) according to the manufacturer’s instructions. RNA quantity and quality were examined using Nanodrop (Thermo Scientific, Waltham, MA, United States) and a Bioanalyzer (Agilent Inc., Santa Clara, CA, United States). cDNA was produced using miRCURY LNA universal RT microRNA PCR assays (Exiqon). MiRCURY LNA™ SYBR Green Master Mix was used for qRT-PCR (Exiqon). All miRNA quantification data were normalized to U6 expression, and all reactions were run on the QuantStudio 12 k Flex system (Applied Biosystems, Foster City, CA) and analyzed by the comparative Ct (ΔΔCt) method (2^−ΔΔCt^ with logarithm transformation). The specific primers used were as follows: miR-760, 5′-CCC​CCT​CAG​TCC​ACC​AGA​G-3′ (F), 5′-GTT​GCA​TTT​CGC​TCC​CCA​C-3′ (R); U6, 5′-CTC​GCT​TCG​GCA​GCA​CA-3′ (F),5′-AACGCTTCACGAATTTGCGT-3′ (R).

### Microarray Analyses and miRNA Target Prediction

Total RNA was extracted with TRIzol reagent (Invitrogen, MA), and then the RNA was purified with an RNeasy Mini kit (Qiagen, Germany). Three pairs of degenerative NP samples and matched controls were tested. Microarray analyses were performed by Biotechnology Corporation (Nanjing, China). Total RNA was Cy-3 labeled and hybridized to miRCURY LNA miRNA arrays for over 16 h at 56°C with Agilent SureHyb-enabled hybridization chambers and a rotating oven. Arrays were then washed, dried, and scanned using an Agilent G2565AA Microarray Scanner System. Array images were analyzed using Agilent Feature Extraction software (version 11.0.1.1). Data were analyzed, and a locally weighted regression filter was then performed to normalize the signals. Significant differentially expressed transcripts were filtered by both the *p*-value and fold-change between the two samples. TargetScanHuman (www.targetscan.org/) and microRNA.org were used to forecast the miRNA target genes and to analyze mRNA binding sites.

### *In Vitro* miRNA Transfection

miR-760 (miRNA mimics/inhibitor) or miRNA negative control (miRNA mimics/inhibitor negative control) labeled with Cy3 were generated using the Silencer^®^ siRNA Labeling Kit (#AM1636) according to the manufacturer’s instructions. Before transfection, the cultured primary human NP cells were seeded at a density of 2 × 10^5^ cells per well in a 24-well plate and then incubated overnight for cell attachment. Then, the NP cells were transfected with mimics, inhibitors, and miRNA negative controls at a final oligonucleotide concentration of 50 nM. All cell transfections were performed using the Lipofectamine RNAiMAX Transfection Reagent (Invitrogen) according to the manufacturer’s instructions. miR-760 mimics, miR-760 inhibitor, and the respective negative controls (NC mimics and NC inhibitor) were synthesized by Life Technologies. The MyD88 expression plasmid (pcDNA3.1/V5-His TOPO TA Expression Kit) was obtained (Invitrogen). The MyD88 expression plasmid was transfected using Lipofectamine PLUS™ reagent (Invitrogen) according to the manufacturer’s instructions. All transfection assays were performed in triplicates. Cells were collected 48 h post transfection for further analysis.

### 3′-UTR Cloning and Luciferase Assay

The psi-CHECKTM-2 vector (Promega, Madison, WI) was used to construct the wild-type MyD88 3′-UTR reporter plasmid (MyD88 3′-UTR). To construct the mutant plasmids, site-directed mutagenesis was performed using the QuikChange Lightning Site-Directed Mutagenesis Kit (Agilent Technologies, Inc., Santa Clara, CA, United States). All constructs were confirmed by sequencing (Cosmo Genetech, Seoul, Korea). The cultured primary NP cells were co-transfected with wild-type or mutant MyD88 3′-UTR-Luc reporter plasmid and microRNA using the Lipofectamine PLUSTM reagent (Invitrogen). Cell lysates were harvested 48 h after transfection, and luciferase activity was assayed using the Dual-Glo luciferase Assay system (Promega, Madison, WI, United States). Relative firefly luciferase activity was normalized to Renilla luciferase activity. All transfection assays were performed in triplicates.

### Flow Cytometry

In order to evaluate the apoptosis, flow cytometry by annexin V-fluorescein isothiocyanate (FITC)/propidium iodide (PI) double staining was conducted according to the manufacturer’s instructions. The cultured primary human NP cells were transfected with mimics, inhibitors, mimics control and inhibitor control at a final oligonucleotide concentration of 50 nM. All cell transfections were performed using the Lipofectamine RNAiMAX Transfection Reagent (Invitrogen) according to the manufacturer’s instructions. The collected cells were washed twice with PBS and centrifuged. The supernatants were discarded and the cells were resuspended in 1 × annexin-binding buffer. Apoptosis was evaluated by staining the cultured primary NP cells with both fluorescein isothiocyanate (FITC) and propidium iodide (PI). Cells that were positively stained with Annexin V-FITC and negatively stained for PI were considered apoptotic. Cells that were positively stained for both Annexin V-FITC and PI were considered necrotic. The experiments were performed in triplicates. After incubation for 15 min at room temperature in the dark, the apoptosis rate of cells was evaluated by FCM using an Epics-XL-MCL flow cytometer (Beckman Coulter).

### 5-Ethynyl-2′-Deoxyuridine Assay

The cultured primary human NP cells were seeded in 24-well plates at a density of 2 × 10^5^ cells per well at 37°C in 5% CO_2_. Cells were treated with 50 μM EdU (Sigma-Aldrich) and incubated for 2 h. Next, the cells were fixed with 4% formaldehyde for 15 min, followed by permeabilization with 0.5% Triton X-100 for 20 min at room temperature (RT). Then, the cells were washed with PBS three times and incubated with 100 μl of 1× Apollo reaction cocktail for 30 min at RT. Subsequently, the cells were stained with Hoechst 33,258. Cell proliferation was analyzed using a fluorescence microscope (Zeiss, Jena, Germany).

### Fluorescence *in Situ* Hybridization

The NP tissues from IDD patients and control patients were used for FISH detection. The sample within paraffin was sliced into films at 4-μm of thickness and attached to slides, which were treated with xylene and a dilution series of ethanol. Subsequently, proteinase K was applied to the slices of tissues under 37°C for 10 min, followed with another dilution series of ethanol. A locked nucleic acid (LNA) probe with complementarity to miR-760 was labeled with 5- and 3-digoxigenin (5′-ATC​GTC​CGT​AGC​TTA​AGA​CTA​CG-3′) and constructed by Exiqon (Woburn, MA, United States). A scrambled LNA probe was used as a negative control (5′-GGC​TAA​CTG​CAT​GCC​AAT​CCG​A-3′).

The slides were prehybridized for 30 min at 52°C and then 10 pmol of the probe in hybridization mixture was added to each slide and incubated for 1 h at 52°C. Slides were incubated in 3% (v/v) hydrogen peroxide (H_2_O_2_) for 10 min at RT. After washing, the slides were incubated with the blocking buffer for 30 min at room temperature. The slides were then treated with an antibody and incubated for 30 min. Fluorescence detection was performed using the TSA Plus Fluorescein System, according to the manufacturer’s protocol. Images were obtained using an FV1000 confocal laser scanning microscope (Olympus IX-81; Olympus, Tokyo, Japan).

### Western Blotting

Cultured primary human NP cells were harvested and lysed in ice-cold radioimmunoprecipitation assay (RIPA) buffer supplemented with a protease inhibitor cocktail and phosphatase inhibitor. Protein concentrations were measured using a BCA protein assay reagent kit (Pierce Biotechnology, Rockford, IL, United States). The prepared protein samples were separated on 10% sodium dodecyl sulfate-polyacrylamide gel (SDS-PAGE) and then transferred to polyvinylidene fluoride (PVDF) membranes at 4°C. The membranes were subsequently blocked with 3% bovine serum albumin (BSA) for 2 h and incubated with primary antibodies and anti-beta-actin in 5% BSA in Tris-buffered saline with Tween 20 (TBS-T) overnight at 4°C, followed by incubation with horseradish peroxidase-labeled secondary antibody. Images were obtained using an Odyssey two-color infrared fluorescence scanning imaging system. The experiment was performed in triplicate. The antibodies used in the western blotting analysis were type II collagen (Col II) (1:500; catalog number: ab34712; Abcam), aggrecan (1:100, catalog number: ab36861; Abcam), matrix metalloproteinase (MMP)-13 (1:100, catalog number: ab39012; Abcam), a disintegrin and metalloproteinase with thrombospondin motifs 5 (ADAMTS5) (1:2,000, catalog number: ab41037; Abcam), β-actin (1:2,000, catalog number: 4,967; Cell Signaling Technology), MyD88 (1:2,000, catalog number: ab133739; Abcam), tumor necrosis factor receptor-associated factor (TRAF)-6 (1:1,000, catalog number: 8,028; Cell Signaling Technology), TGF-β-activated kinase 1 (TAK1) (1:3,000, catalog number: ab109526; Abcam), p65 (1:3,000, catalog number: ab32536; Abcam), and p-p65 (1:500, catalog number: ab278777; Abcam).

### Cell Immunofluorescence

The cultured primary human NP cells were fixed with 4% paraformaldehyde for 20 min, rinsed with PBS containing 0.25% Triton X-100 for 10 min, and then blocked with 4% BSA, containing 0.25% Triton X-100, for 1–2 h at 37°C. The cells were incubated with primary antibodies against Col II (1:2,000, catalog number: ab34712; Abcam) and MMP13 (1:200, catalog number: ab39012; Abcam) overnight at 4°C. After rinsing, the cells were incubated with the corresponding secondary antibody (1:500, catalog number: ab150078; Abcam). The fluorescence images were visualized under a confocal microscope (LSM710; Carl Zeiss, Oberkochen, Germany) and analyzed using Image-Pro Plus 6.0.

### Immunofluorescent Staining of Histological Sections and Terminal Deoxyribonucleotidyl Transferase-Mediated dUTP Nick-End Labeling Staining

Tissues of mouse discs were fixed with 4% paraformaldehyde for 5 min, washed with TBS-T. Intervertebral discs were decalcified, and embedded in 10% formalin,10% EDTA, and paraffin. Sagittal sections of disc tissue were then cut every 3 μm from the midsagittal plane. Paraffin-embedded slices were deparaffinized and treated with 1 mM ethylenediaminetetraacetic acid (EDTA) at 80°C for 15 min. The slices were then incubated with 10 mg/ml hyaluronidase at 37°C for 30 min. The slices were blocked with bovine serum albumin and incubated with primary antibodies against Col II (1:2,000, catalog number: ab34712; Abcam) and MMP13 (1:200, catalog number: ab39012; Abcam) for 2 h. Paraffin-embedded slices were deparaffinized and treated with 1 mM ethylenediaminetetraacetic acid (EDTA) at 80°C for 15 min. The sections were then incubated with 10 mg/ml hyaluronidase at 37°C for 30 min. The slices were then hybridized with secondary antibodies and labeled with 4′, 6-diamidino-2-phenylindole (DAPI) (Invitrogen). Fields were acquired using fluorescence microscopy (Nikon, Japan), and ImageJ software (Bethesda, United States) was used for fluorescence intensity quantification. To detect NP cell apoptosis, TUNEL assay was performed according to the manufacturer’s instructions (*In Situ* Cell Death Detection Kit, Fluorescein; Roche).

### Statistical Analysis

All data analyses were conducted using SPSS software (version 21.0; IBM Corp., Armonk, NY, United States). All continuous data were tested for normal distribution by using the Kolmogorov-Smirnov test. The Mann–Whitney *U* test was used to analyze the qRT-PCR results. The Pearson’s correlation test was employed to evaluate the associations between the expression of miR-760 and disc degeneration grade of patients. A chi-square test was used to analyze the sex distribution difference between the two groups. Statistical analysis comparing multiple groups with one-way analysis of variance followed by Tukey’s post hoc. Statistical significance was set at *p* < 0.05.

## Results

### Discovery of miRNAs Differentially Expressed in Degenerative NP Cells by Microarray Analysis

**FIGURE 1:**
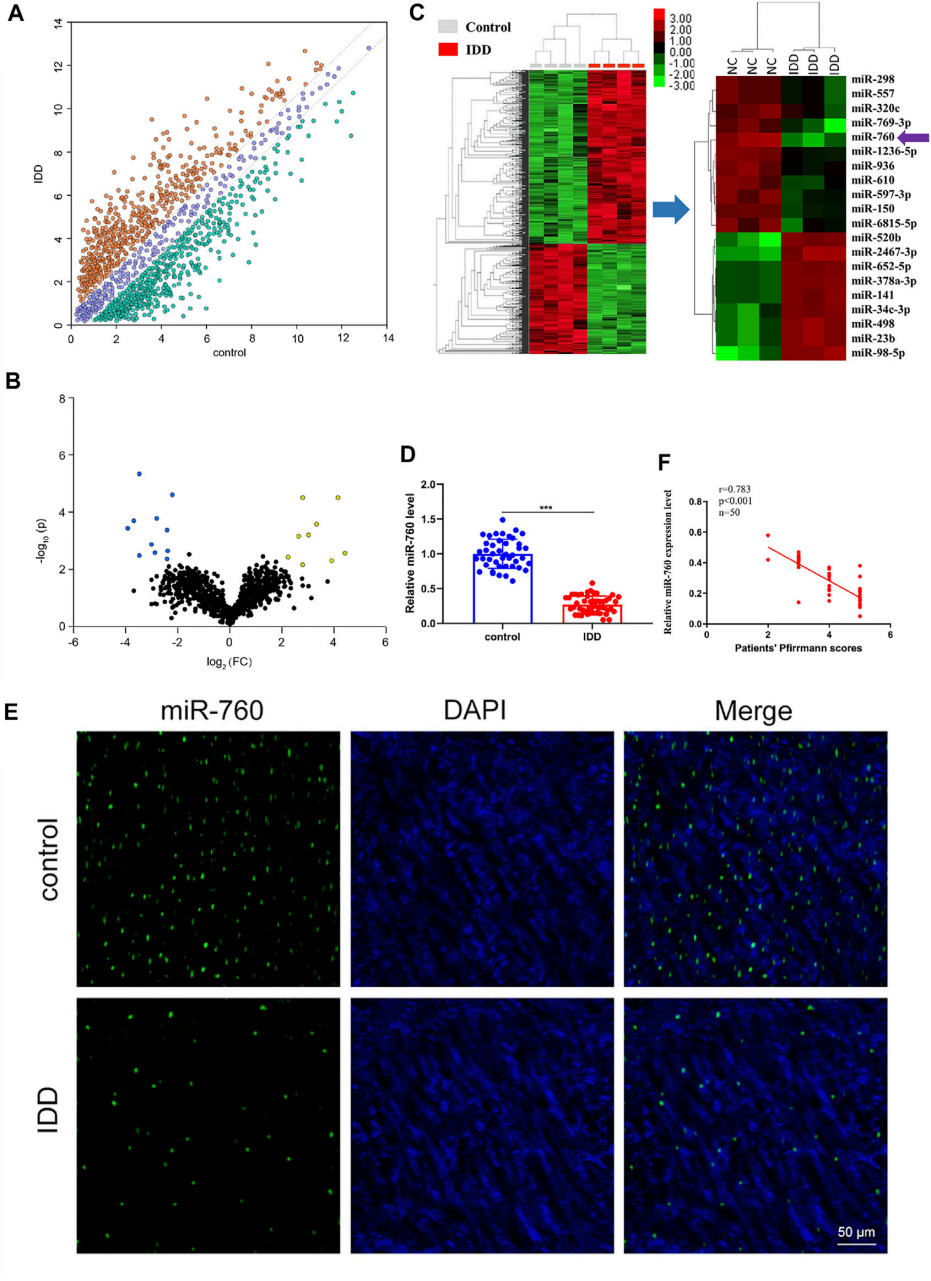
Identification of differentially expressed miRNAs in NP tissues of IDD patients. **(A)** Scatter plot of miRNA expression profile between IDD patients and controls (yellow dots, up-regulation more than two-fold; green dots, down-regulation more than two-fold). **(B)** The biological and statistical significance of differently expressed miRNAs between IDD patients and controls were illustrated by Volcano plot. Yellow dots indicated the upregulated (right side) and blue dots indicated downregulated (left side) miRNAs. MiR-760 was indicated. **(C)** The heat maps of differently expressed miRNAs in IDD patients. The 11-down regulated and nine up-regulated miRNAs with the most obvious change were shown. **(D)** Compared with the control group (n = 43), miR-760 expression level was down-regulated in IDD patients (n = 50). ***p < 0.01 by Mann–Whitney *U* test. **(E)** FISH analysis of NP tissues from IDD patients demonstrated that the level of miR-760 was decreased. **(F)** The expression of miR-760 in NP cells from IDD patients was negatively correlated with the disc degeneration grade (n = 50; r = 0.783, *p* < 0.001).

To investigate the dysregulated miRNAs in IDD, microarray analysis of human NP tissues (three IDD patients vs. three normal controls) was performed (Figure 1A). Patients with IDD could be distinguished from the controls by the significantly dysregulated miRNAs shown in unsupervised clustering analysis (Figures 1B,C). We identified nine upregulated and eleven downregulated miRNAs that exhibited a mean fold change of more than 2-fold or less than 0.5-fold as well as a *p*-value less than 0.05. RT-PCR assay was used to confirm the expression of dysregulated miRNAs. In the training set, the 20 candidate miRNAs were measured in a separate set of samples from 6 patients with IDD and 6 controls. Further validation was performed only when the miRNAs met the following criteria: mean fold change >2.0 or <0.5 and *p* values <0.01. According to the criteria, miR-760, miR-150, miR-652-5p, and miR-141 were selected for further validation (Table 1). In the validation set, the concentration of the four dysregulated miRNAs were measured by RT-PCR in a larger cohort comprising of 50 patients and 43 controls. The miR-760 expression pattern was confirmed to be the most significantly downregulated in the NP tissues and selected as the candidate miRNA for further study (Figure 1D). The decreased level of miR-760 was further confirmed by FISH (Figure 1E). Moreover, the expression of miR-760 in NP cells from IDD patients was negatively correlated with the disc degeneration grade (n = 50; r = 0.783, *p* < 0.001; Figure 1F).

**TABLE 1:** Differentially expressed miRNAs in NP tissues from IDD and controls.

miRNAs	Training Set		Validation Set	
	Fold Change *P*		Fold Change *P*	
Down-Regulated				
Hsa-miR-298	0.23	0.09	—	—
Hsa-miR-557	0.17	0.34	—	—
Hsa-miR-320c	0.38	0.15	—	—
Hsa-miR-769-3p	0.03	0.47	—	—
** Hsa-miR-760**	**0.06**	0.005**	**0.09**	0.001**
Hsa-miR-1236-5p	0.41	0.06	—	—
Hsa-miR-936	0.12	0.27	—	—
Hsa-miR-610	0.35	0.08	—	—
Hsa-miR-597-3p	0.22	0.41	—	—
** Hsa-miR-150**	**0.13**	0.002**	**0.17**	**0.39**
Hsa-miR-6815-5p	0.36	0.53	—	—
Up-regulated				
Hsa-miR-520b	7.9	0.23	—	—
Hsa-miR-2467-3p	9.3	0.08	—	—
** Hsa-miR-652-5p**	**6.4**	0.003**	**5.7**	**0.38**
Hsa-miR-378a-3p	10.9	0.07	—	—
** Hsa-miR-141**	**15.2**	0.007**	**13.9**	**0.16**
Hsa-miR-34c-3p	9.8	0.42	—	—
Hsa-miR-498	4.5	0.29	—	—
Hsa-miR-23b	8.4	0.31	—	—
Hsa-miR-98-5p	11.7	0.56	—	—

NP, nucleus pulposus; IDD, intervertebral disc degeneration; Hsa: human; **p < 0.01.

### Effects of miR-760 Overexpression or Silencing on the Phenotypes of NP Cells

**FIGURE 2:**
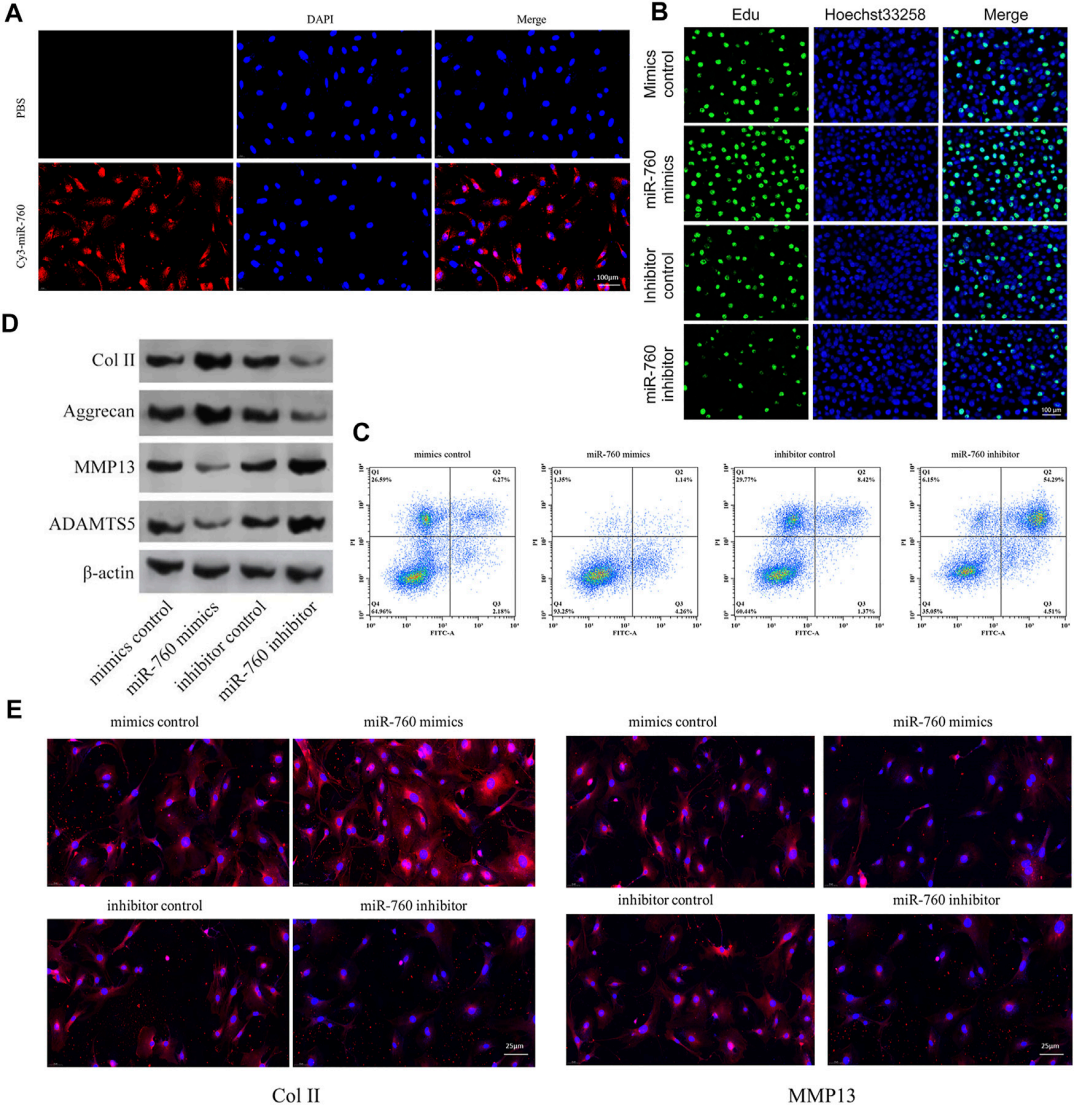
*In vitro in vitro* study of miR-760. **(A)** Cy3 confirmed that miR-760 was transfected into primary cultured human NP cells. Scale bar = 100 μm. **(B)** Cell proliferation was analyzed in miR-760 mimics or inhibitor transfected cultured primary human NP cells using EdU assays. n = 3 replicates per group. Scale bar = 100 μm. **(C)** Analysis of NP cells apoptosis was assayed by FCM. n = 3 replicates per group. **(D)** The expression levels of collagen II, aggrecan, MMP13, and ADAMT5 were detected by western blot. **(E)** The expression levels of collagen II and MMP13 were detected by the immunofluorescence. Scale bar = 25 μm.

The functional effect of miR-760 on cell proliferation, cell apoptosis and matrix synthesis *in vitro* was assessed by transfecting primary cultured human NP cells with miR-760 mimics or inhibitors to induce or repress the expression of miR-760. Successful transfection of miR-760 in NP cells was verified using Cy3-labeled miRNAs (Figure 2A). miR-760 mimics-treated NP cells exhibited higher proliferative capacities than the mimic control as shown by the EdU assay, whereas miR-760 inhibitor transfection dramatically inhibited the proliferation of NP cells (Figure 2B). Meanwhile, overexpression of miR-760 led to a decrease in NP cell apoptosis (Figure 2C). Gain-of-function and loss-of-function assays were performed to confirm whether miR-760 could functionally regulate collagen synthesis in NP cells. Compared with the controls, administration of miR-760 mimics increased the expression levels of collagen II and aggrecan. In contrast, NP cells transfected with the miR-760 inhibitor significantly increased the levels of MMP13 and ADAMTS-5 (Figure 2D). These results were further confirmed by immunofluorescence analysis (Figure 2E). These findings collectively demonstrate that the miR-760 profile substantially affects the phenotypes of NP cells.

### Identification of *MyD88* as a Target Gene for miR760

**FIGURE 3:**
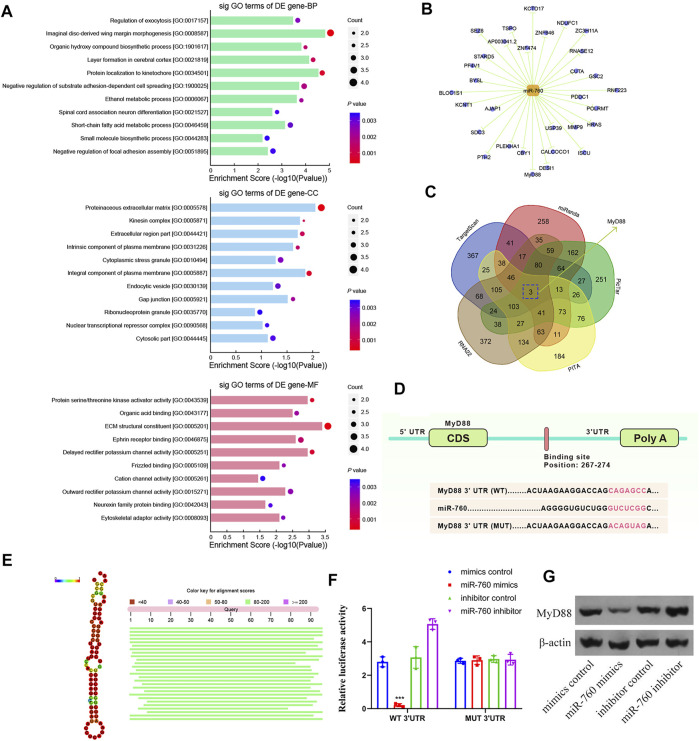
Identification of MyD88 as a target of miR-760. **(A)** Downregulated GO terms with the most significant *p* values for biological processes, molecular function, and cellular component. **(B)** Cytoscape was used to confirm the target of miR-141. **(C)** Venn diagram displaying miR-760 computationally predicted to target MyD88 by five different prediction algorithms: miRanda, TargetScan, RNA22, PicTar, and PITA. **(D)** Sequence alignment of a putative miR-760-binding site within the 3′UTR of MyD88 mRNA shows a high level of sequence conservation and complementarity with miR-760. **(E)** High conservation of miR-760. **(F)** The wild- or mutant-type MyD88 3′UTR reporter plasmid was co-transfected with miR-760 mimics or inhibitor into cultured primary human NP cells. Luciferase activity was measured after transfection. n = 3 replicates per group, ***p < 0.001 by one-way ANOVA test followed by Tukey’s post hoc. **(G)** MyD88 expression level was detected by western blot. n = 3 replicates per group.

To elucidate the molecular mechanism of miR-760 in the regulation of IDD, a series of assays were performed to determine the target mRNA transcripts. All the dysregulated miRNAs detected by microarrays of human NP tissues (3 IDD patients vs Three normal controls) were identified and subjected to gene ontology (GO) analysis. GO analysis of the dysregulated genes with the most significant *p* values for biological processes, molecular functions, and cellular components were associated with imaginal disc-derived wing margin morphogenesis (GO: 0008587), ECM structural constituents (GO: 0005201), and extracellular regions (GO: 0044421) (Figure 3A). Cytoscape was used to construct the miRNA–mRNA network (Figure 3B). A Venn diagram displaying miR-760 was computationally predicted to target MyD88 using different algorithms (Figure 3C). Notably, the 3′-UTR of MyD88 mRNA included a putative miR-760-binding site that showed a high level of sequence conservation (Figures 3D,E). To validate whether MyD88 was directly downstream of miR-760, we performed luciferase reporter assays with a MyD88 vector that contained either the putative miR-760 binding sites (wild type) or the mutant binding sites (MUT). Delivery of miR-760 mimics significantly inhibited the relative luciferase reporter activity, while the miR-760 inhibitor enhanced the reporter activity in NP cells (Figure 3F). Furthermore, mutation of the miR-760 binding sites within the MyD88 3′-UTR abolished the repressing effects of miR-760. The repressing effect was also validated by protein expression in NP cells (Figure 3G). Taken together, these findings indicate that miR-760 acts as a direct suppressor of MyD88 in NP cells.

### miR-760 Regulates IDD by Modulating the MyD88/NF- κB Signaling Pathway

**FIGURE 4:**
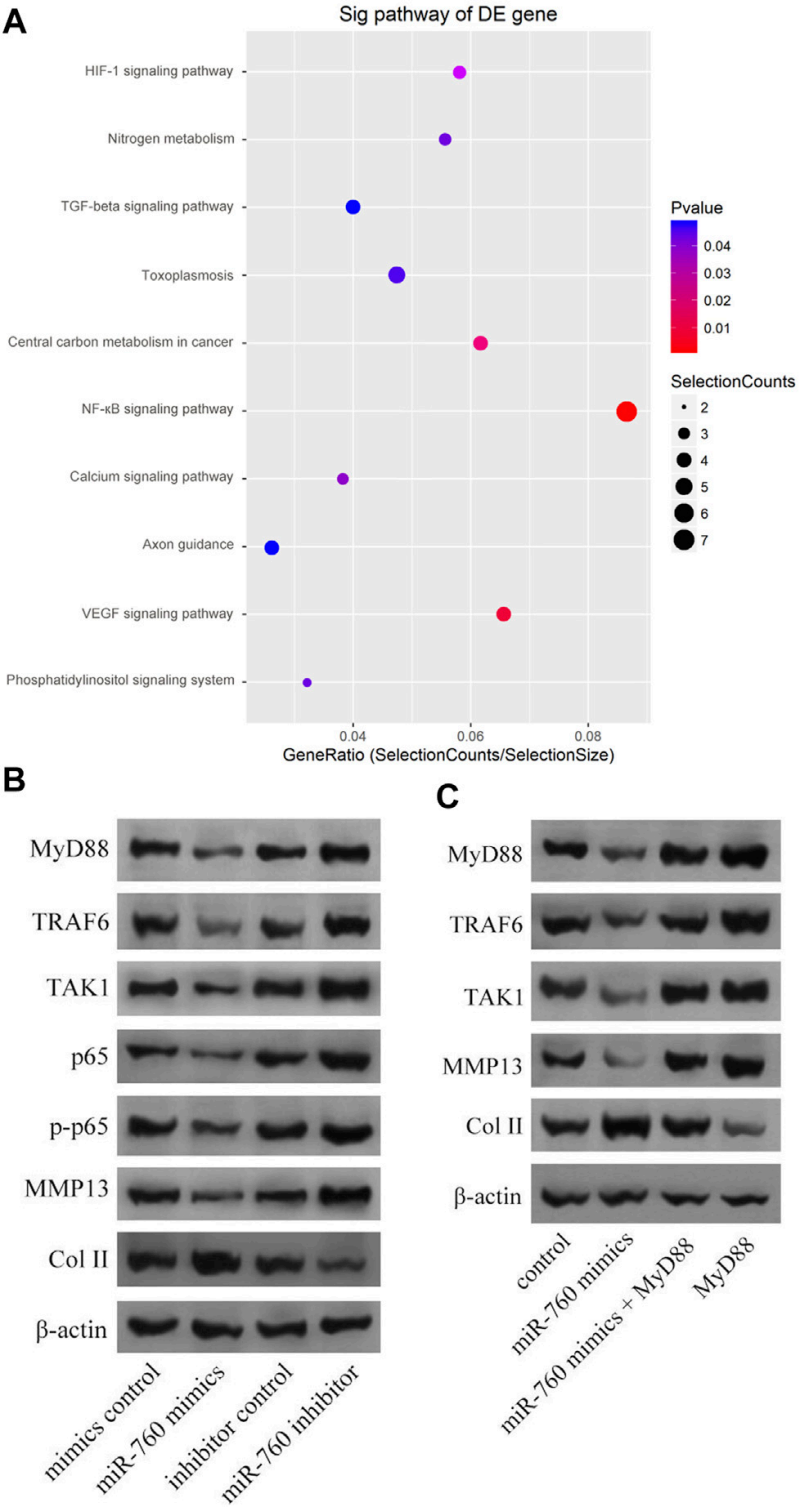
MiR-760 exerted its functions by targeting the MyD88/κB signaling pathway. **(A)** KEGG analysis demonstrated that NF-κB signaling pathway enriched in IDD. **(B)** Cultured primary normal human NP cells were transfected with miR-760 mimics, miR-760 inhibitor, their negative control and then the levels of MyD88, TRAF6, TAK1, p65, p-p65, MMP13 and Col II were measured by western blotting. n = 3 replicates per group. **(C)** The rescue experiments were performed in cultured primary human NP cells to validate the relationship between miR-760 and MyD88. Inhibition of TRAF6, TAK1 and MMP13 expression levels by miR-760 mimics was rescued by restoration of MyD88 expression. In comparison, inhibition of Col II expression levels by MyD88 overexpression was rescued by miR-760 mimics. n = 3 replicates per group.

Kyoto Encyclopedia of Genes and Genomes analysis demonstrated that the NF-κB signaling pathway was enriched in IDD (Figure 4A). Moreover, MyD88 acts as an activating factor in the NF-κB signaling pathway (Vijayan et al., 2014; Zhang et al., 2014). The discovery that downregulation of miR-760 was associated with IDD led us to investigate the potential association between miR-760 and the MyD88/NF-κB signaling pathway. Cultured human NP cells were transfected with miR-760 mimic, miR-760 inhibitor, or miR-Control. MiR-760 mimics significantly downregulated the expression of NF-κB signaling pathway dependent proteins, including TRAF6, TAK1, p65, p-p65, and MMP13, whereas the miR-760 inhibitor significantly upregulated the expression of these proteins (Figure 4B). Rescue experiments were also performed in cultured primary human NP cells to confirm the relationship between miR-760 and MyD88. Restoration of MyD88 expression rescued the inhibition of MyD88, TRAF6, TAK1, and MMP13 expression levels induced by the overexpression of miR-760, whereas the inhibition of Col II expression levels by MyD88 was rescued by the restoration of miR-760 mimic expression (Figure 4C). These results suggest that downregulation of miR-760 was associated with IDD through the MyD88/NF-κB pathway.

### Therapeutic Use of miR-760 in an IDD Mouse Model

**FIGURE 5:**
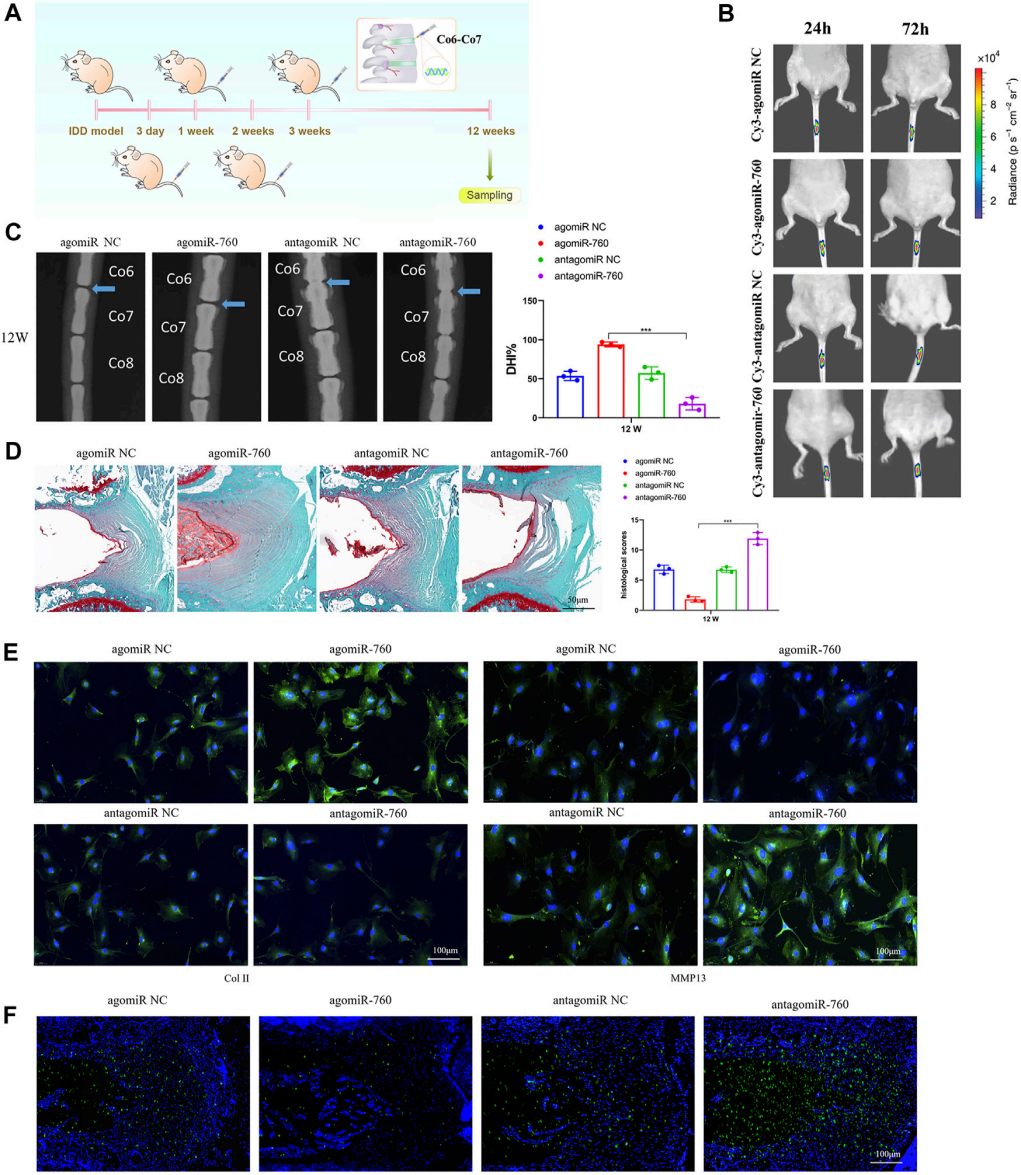
Local injection of agomiR-760 attenuated IDD development. **(A)** Overview of the experimental set-up with deliveries of agomiR-760, agomiR NC, antagomiR-760 or antagomiR NC at 3, 7, 14, 21 days postoperatively. **(B)** Fluorescence image in mice at 24 and 72 h after the administration of Cy3-agomiR-760 showed that the delivery was ideal. The color bar (from blue to red) indicated the change in fluorescence signal intensity from low to high. **(C)** Radiological outcomes of intervertebral disc degeneration was evaluated by X-ray. A significant increase in DHI% was noted in mice treated by agomiR-760 at 12 weeks after surgery. ***p < 0.001 by one-way ANOVA test followed by Tukey’s post hoc. **(D)** Histological outcomes of intervertebral disc degeneration was evaluated by Safranin O staining. The cells of the NP region in the intervertebral disc were more abundant in mice treated with agomiR-760. Mice treated by agomiR-760 showed a significant decreased histological score. ***p < 0.001 by one-way ANOVA test followed by Tukey’s post hoc. Scale bar = 50 μm. **(E)** Immunostaining showed that agomiR-760 remarkably increased the expression of collagen II, while the expression of MMP 13 was decreased. Scale bar = 100 μm.**(F)** TUNEL staining demonstrated that mice treated with agomiR-760 showed significantly decreased NP cell apoptosis. Scale bar = 100 μm.

To investigate the therapeutic effect of miR-760 on IDD and to elucidate its potential molecular mechanisms, we induced IDD in WT mice, and performed local injection of agomiR-760, agomiR NC, antagomiR-760, or antagomiR NC at 3, 7, 14, and 21 days postoperatively (Figure 5A). The fluorescence images in mice showed that the delivery was ideal (Figure 5B). Radiological and histological outcomes confirmed that the IVDs with local delivery of agomiR-760 showed significantly higher DHI% and lower histological scores, indicating that the IVDs were effectively protected. In contrast, severe disc degeneration developed in the mice treated with antagomiR-760 (Figures 5C,D). Moreover, agomiR-760 remarkably increased the expression of Col II, while the expression of MMP 13 was decreased. Mice treated with antagomiR-760 showed the opposite results (Figure 5E). TUNEL staining demonstrated that mice treated with agomiR-760 showed significantly decreased NP cell apoptosis (Figure 5F). In conclusion, the results indicated that miR-760 had a therapeutic effect in protecting the IVDs from destruction.

## Discussion

As key regulatory factors of gene expression, miRNAs have been implicated in the prognosis and progression of many diseases, and may be used as potential targets for gene therapy (Gregory et al., 2008; Mateescu et al., 2011; Wang et al., 2021). Recent studies have shown that miRNAs are dysregulated in NP cells in patients with IDD (Jiang et al., 2021a; Jiang et al., 2021b). This has fueled interest in exploring their functions and potential as novel therapeutics. In the present study, miR-760 expression levels were found to be downregulated in degenerative NP tissues and negatively correlated with the degree of disc degeneration. Subsequent *in vitro* assays showed effects of miR-760 on cell proliferation, matrix synthesis and proteases expression. *In vivo* studies also suggested the overexpression of miR-760 could exert a potential therapeutic influence on the development of IDD.

MyD88 is a signal adaptor molecule that links toll-like receptors and interleukin-1 receptors with downstream signaling molecules, and exerts its function in immune and inflammatory responses (Ellman et al., 2012). Studies have shown that the MyD88-mediated signaling pathway is closely related to the pathogenesis of IDD (Qin et al., 2016; Zhang et al., 2018). In this study, a sequence complementary to miR-760 was identified at the 3′-UTR of MyD88 mRNA, and overexpression of miR-760 significantly decreased the expression of MyD88. Transfection of NP cells with miR-760 mimics decreased the luciferase reporter activity, while it was significantly increased in NP cells transfected with the miR-760 inhibitor. Moreover, the luciferase activity of MUT MyD88-3′-UTR vector did not affect the co-transfection with the miR-760 mimic, indicating that miR-760 directly interacted with the 3′-UTR of MyD88 to inhibit the translation of chimeric transcripts.

Previous studies have indicated that the activation of MyD88 activates the NF-κB signaling pathway (Vijayan et al., 2014; Zhang et al., 2014). The canonical NF-κB pathway, which is known for its crucial role in the regulation of a range of catabolic processes in response to aging, inflammation, and cell injury, also plays a pivotal role in IDD. Nasto et al. (Nasto et al., 2012) reported that the activation of NF-κB is positively correlated with the degeneration grade in the mouse IVDs. NF-κB plays important roles in regulating the expression of IL-1β, IL-6, TNF-α, MMPs, and other inflammatory factors that could damage the structural proteins of IVDs, such as Col II. In this study, NP cells transfected with miR-760 mimics showed decreased expression of MyD88, TRAF6, TAK1, p65, p-p65, and MMP13, while these factors were significantly upregulated when NP cells were transfected with the miR-760 inhibitor. Upregulation of Col II upon overexpression of miR-760 was significantly attenuated by the re-introduction of MyD88. The results indicated that miR-760 regulated IDD by targeting the MyD88/NF-κB pathway.

The results of this study provided a new perspective for the clinical translation of miR-760 as a potential diagnostic and treatment strategy for IDD. The significantly decreased expression of miR-760 detected in IDD patients signified that miR-760 could be used as a stable biomarker for IDD. The *in vitro* study revealed that with the upregulation of miR-760, the expression of MyD88 was downregulated and the NF-κB signaling pathway was suppressed, resulting in the overexpression of extracellular matrix protein (Col II) and decreased expression of extracellular matrix-degrading enzymes (MMP13). *In vivo* experiments, local delivery of agomiR-760 NPs could decrease the apoptosis of NP cells and exert a therapeutic influence on the development of IDD. On the contrary, the IDD mice treated with antagomiR-760 showed aggravated disc degeneration. All these findings indicated that miR-760 was a molecular regulator in the pathogenetic mechanism of IDD and a new potential option for the gene therapy of patients with IDD.

In additions to the study’s important findings, limitations on the scope of research still exist. First, the microarray analysis of human NP tissues was performed for only three IDD patients and three normal controls. It might not be enough to draw strong conclusions on the miRNA profile. Second, the control samples were obtained from patients with fresh traumatic lumbar fractures who underwent anterior decompressive surgery. The samples in the control group might have some damage, resulting in a bias for the interpretation of the miRNA profile. Third, only twelve mice were included in the *in vivo* experiment, a n = 3 mice per group seemed underpowered to reach the conclusions. Further studies with more mice were needed to validate the results.

In this study, miR-760 was found to be significantly downregulated in IDD NP cells. Subsequent *in vitro* studies revealed that high levels of miR-760 might inhibit IDD by targeting the MyD88/NF-κB signaling pathway. The *in vitro* and *in vivo* results indicated that miR-760 had a potent protective effect against IDD, which may be beneficial for the clinical therapy of patients with IDD.

## Data Availability

The datasets presented in this study can be found in online repositories. The names of the repository/repositories and accession number(s) can be found below: ArrayExpress (Number: E-MTAB-11554).
